# Supplementary material for: Physicochemical Properties and Elimination of the Activity of Anti-Nutritional Serine Protease Inhibitors from Mulberry Leaves
Source: Molecules. 2022 Mar 11;27(6):1820. doi: 10.3390/molecules27061820 (PMC8948906; doi:10.3390/molecules27061820)
Supplement: Supplementary file 1 [file molecules-27-01820-s001.zip › molecules-1614295-supplementary.pdf]

Suppementary Material

Physicochemical Properties and Elimination of the Activity of Anti-Nutritional Serine Protease Inhibitors from Mulberry Leaves

Zhuxing Luo [1], Jinhong Yang [2], Jie Zhang [1], Gang Meng [2], Qingjun Lu [1], Xi Yang [1], Ping Zhao [3] and Youshan Li [1,*]

[1] College of Biological Science and Engineering, Shaanxi University of Technology, Hanzhong 723001, China; luozhx@snut.edu.cn (Z.L.); zhangjie@snut.edu.cn (J.Z.); luqj@snut.edu.cn (Q.L.); yangxi@snut.edu.cn (X.Y.)

[2] Shaanxi Key Laboratory of Sericulture, Ankang University, Ankang 725099, China; yangjinhong@aku.edu.cn (J.Y.); nsymg@aku.edu.cn (G.M.)

[3] State Key Laboratory of Silkworm Genome Biology, Southwest University, Chongqing 400715, China; zhaop@swu.edu.cn

* Correspondence: li_youshan@snut.edu.cn or li_youshan@126.com

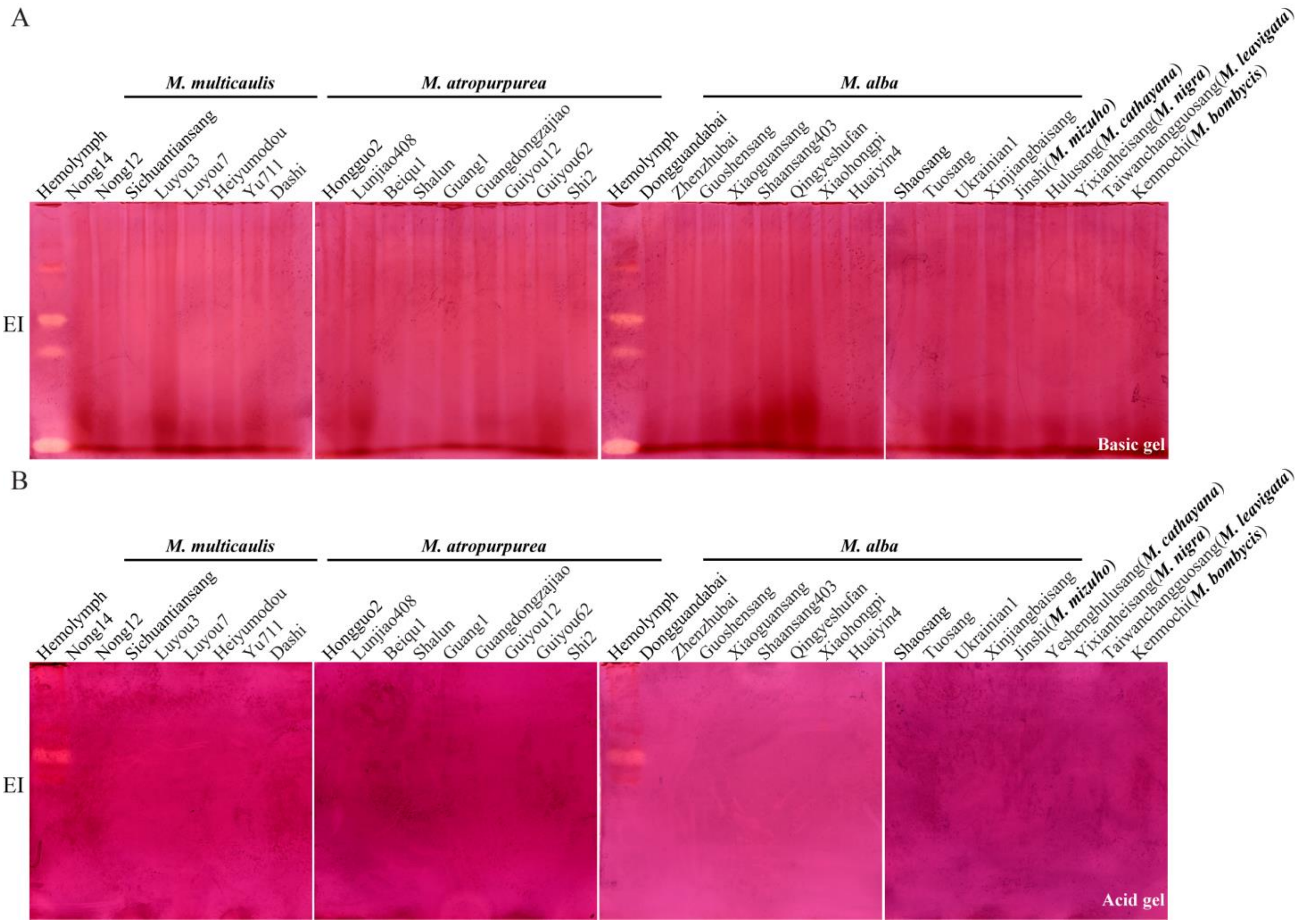

Figure S1. Activity staining of EIs from leaves of different mulberry varieties. Activity staining of EIs from mulberry leaves based on alkaline (A) and acidic (B) Native PAGE. “Basic gel” or “Acid gel” indicate Native PAGE under alkaline or acidic conditions, respectively. “EI” represents elastase inhibitor. B. mori hemolymph from day-5 fifth-instar larvae was used as positive control. From left to right, varieties from “Nong14” to “Yu711” belong to M. multicaulis; varieties from “Dashi” to “Shi2” belong to M. atropurpurea; varieties from “Dongguangdabai” to “Xinjiangbaisang” belong to M. alba. The last five mulberry varieties belong to M. mizuho, M. cathayana, M. nigra, M. leavigata and M. bombycis, respectively.

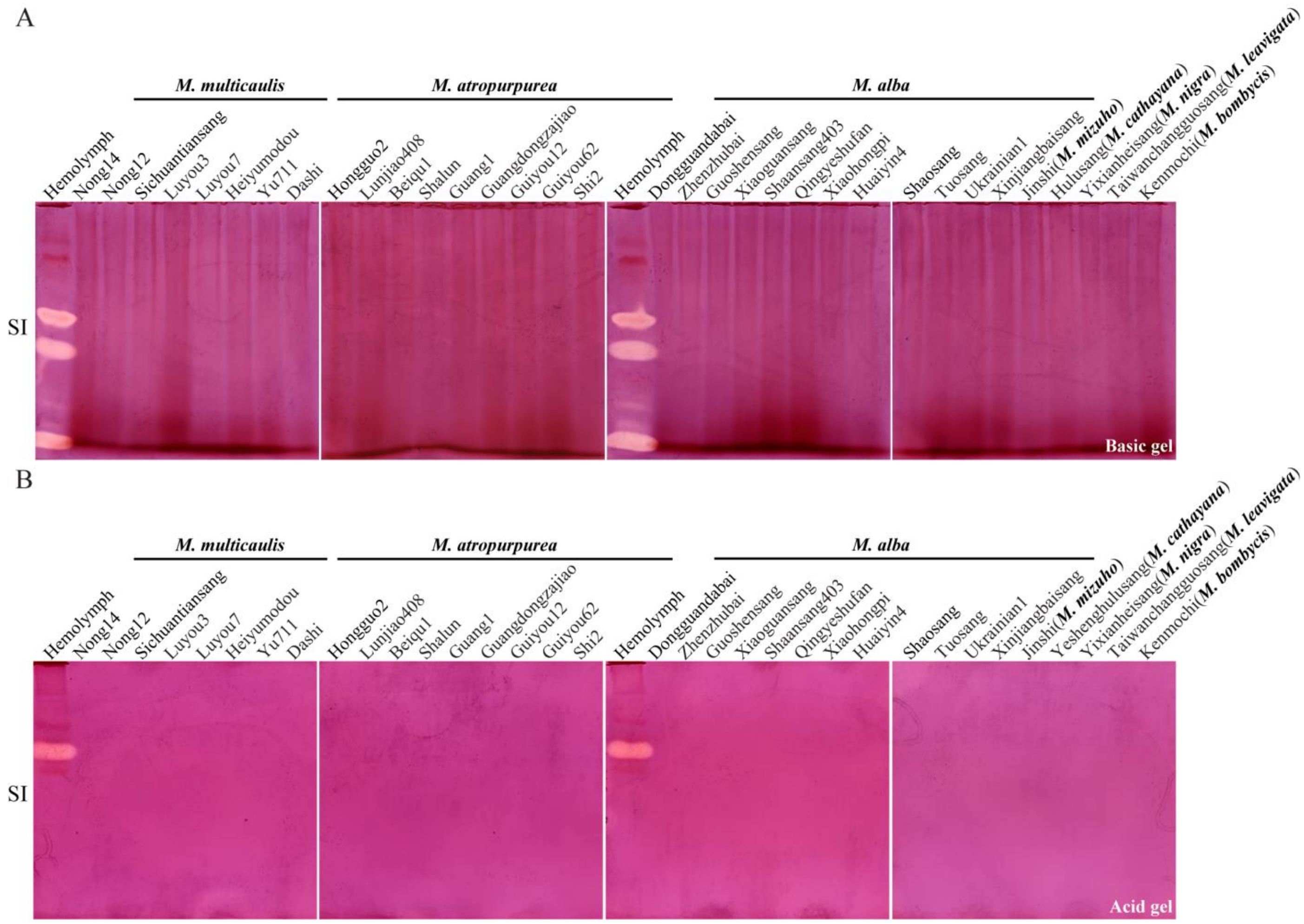

Figure S2. Activity staining of SIs from leaves of different mulberry varieties. Activity staining of SIs from mulberry leaves based on alkaline (A) and acidic (B) Native PAGE. "Basic gel" or "Acid gel" indicate Native PAGE under alkaline or acidic conditions, respectively. "SI" represents subtilisin inhibitor. B. mori hemolymph from day-5 fifth-instar larvae was used as positive control. From left to right, varieties from "Nong14" to "Yu711" belong to M. multicaulis; varieties from "Dashi" to "Shi2" belong to M. atropurpurea; varieties from "Dongguangdabai" to "Xinjiangbaisang" belong to M. alba. The last five mulberry varieties belong to M. mizuho, M. cathayana, M. nigra, M. leavigata and M. bombycis, respectively.
